# Photoactivated *Hypericum perforatum*-Derived Exosome Nanovesicles Suppress Breast Cancer via miR-172f/CD24–Siglec-G/10-Driven Macrophage Reprogramming

**DOI:** 10.34133/bmr.0393

**Published:** 2026-07-17

**Authors:** Peiyuan Zeng, Jiaojiao Lei, Yu Lu, Wenyu Zhang, Jingcan You, Youkun Zheng, Jianbo Wu

**Affiliations:** ^1^ Basic Medicine Research Innovation Center for Cardiometabolic Diseases, Ministry of Education, Southwest Medical University, Luzhou 646000, China.; ^2^ Laboratory for Cardiovascular Pharmacology, Department of Pharmacology, School of Pharmacy, Southwest Medical University, Luzhou 646000, China.; ^3^ Luzhou Municipal Key Laboratory of Thrombosis and Vascular Biology, Southwest Medical University, Luzhou 646000, China.

## Abstract

Photodynamic therapy is traditionally based on reactive-oxygen-species-mediated tumor cell killing, but its immunomodulatory capacity is not fully explored. Plant-derived exosome-like nanovesicles provide a natural photosensitizer platform with potential for dual tumoricidal and immune-modulating effects. This study investigated whether light activation of *Hypericum perforatum*-derived exosome nanovesicles (HPDENs) remodels their microRNA cargo to enhance antitumor activity and reprogram the tumor immune microenvironment. Photoactivation significantly up-regulated miR-172f-p3 in HPDENs, which directly targeted CD24 messenger RNA and suppressed its expression in MCF-7 cells. CD24 down-regulation reprogrammed co-cultured M2 macrophages toward an M1 phenotype by attenuating the CD24–Siglec-G/10 axis. In vivo, light-treated HPDENs and miR-172f-p3 mimics inhibited tumor growth, promoted M1 polarization, and displayed excellent safety with no observable systemic toxicity. Photoactivated HPDENs exert dual antitumor effects by inducing direct tumor suppression and remodeling the immune microenvironment through miR-172f-p3/CD24–Siglec-G/10 signaling. This strategy offers a novel immunotherapeutic avenue that synergizes with photodynamic therapy, supporting the development of plant-derived nanovesicles as next-generation cancer therapeutics.

## Introduction

Breast cancer remains a major cause of morbidity and mortality worldwide despite significant progress in surgery, chemotherapy, targeted therapies, and immunotherapy [1]. Photodynamic therapy (PDT) offers spatially controlled tumor ablation and has seen renewed interest when combined with immunomodulatory approaches [2,3]. Classical PDT focuses on reactive oxygen species (ROS) production and direct tumor cell killing; however, PDT can also remodel the tumor microenvironment and interact with innate immune checkpoints, suggesting opportunities for combined photochemical–immunotherapy approaches [4,5].

Plant-derived extracellular vesicle-like nanoparticles (hereafter “HPDENs” for *Hypericum perforatum*-derived exosome-like nanovesicles) are emerging as biocompatible carriers of natural photosensitizers and nucleic acid cargo, including microRNAs (miRNAs) that can cross species barriers and influence mammalian gene expression [6–8]. *Hypericum* species contain potent photosensitizers such as hypericin that confer intrinsic photodynamic activity. Our prior work established HPDENs as an effective natural photosensitizer for tumor PDT [9]. Yet it remained unknown whether light exposure alters the molecular cargo of HPDENs—particularly miRNA content—and whether such changes contribute to immune modulation beyond direct ROS-mediated cytotoxicity.

In this study, we test the hypothesis that light pre-treatment reprofiles HPDEN miRNA cargo, enriching specific miRNAs that target tumor immune checkpoints. We focused on nta-miR-172f-p3 (abbreviated as miR-172f-p3) because our sequencing revealed its substantial up-regulation after light exposure and because bioinformatics prediction suggested CD24 as a conserved target. CD24–Siglec-10/G is an innate “don’t eat me” checkpoint implicated in macrophage suppression and tumor immune evasion [10–14]. Macrophages within the tumor microenvironment exhibit plasticity, polarizing into either antitumorigenic M1 phenotypes or protumorigenic M2 phenotypes [15,16]. Inducing M1 polarization is a crucial strategy for reprogramming the immunosuppressive tumor microenvironment. We therefore evaluated whether photoactivated HPDENs enhance antitumor immunity by down-regulating tumor CD24 via exosomal miR-172f-p3 and thereby promoting macrophage M1 polarization.

## Materials and Methods

### Cell culture

RAW264.7 murine macrophages and MCF-7 human breast cancer cells were maintained under standard incubation conditions (37 °C, 5% CO_2_). RAW264.7 cells were expanded in Dulbecco’s modified Eagle medium (DMEM) supplemented with 10% fetal bovine serum (FBS) and antibiotics. To generate polarized macrophage subsets, unstimulated RAW264.7 cells were exposed for 24 h to lipopolysaccharide (10 ng/ml) combined with tumor necrosis factor-α (TNF-α; 15 ng/ml) to induce an M1-like phenotype, or to interleukin-4 (IL-4; 20 ng/ml) to promote M2 differentiation.

MCF-7 cells obtained from the China Center for Type Culture Collection were propagated in DMEM containing 10% FBS and antibiotics.

### Extraction of HPDENs

To improve yield and purity, the standard extraction protocol [9,17] was modified with a postcentrifugation ultrafiltration step. This modification yielded a 15% increase in nanoparticle concentration, as measured by NanoSight NS300. A comprehensive physicochemical characterization of these specific HPDENs was recently detailed by our group [9]. Physical characterization included nanoparticle tracking analysis (NTA) for particle size distribution and zeta potential measurements to assess surface charge. Light-treated and non-light-treated HPDENs were compared to assess changes induced by photoactivation.

### Photoactivation protocol

Fresh HPDEN aliquots (normalized by particle number) were placed in sterile, thin-walled clear tubes and irradiated with a 590-nm light-emitting diode (44 mW/cm^2^) for 30 min at room temperature in a cooling chamber. Sham-treated HPDENs were handled identically but kept in the dark. Immediately following irradiation, samples were placed on ice and processed for downstream assays (RNA extraction, cell treatment, or storage at −80 °C).

For in vitro experiments, HPDENs were photoactivated prior to incubation with MCF-7 cells as described above (590 nm, 44 mW/cm^2^, 30 min). No direct light irradiation was applied to cells unless otherwise specified.

### Western blotting

Protein lysates were prepared from cells or tissues and quantified prior to electrophoresis. Equal protein amounts were resolved by sodium dodecyl sulfate–polyacrylamide gel electrophoresis and transferred onto polyvinylidene fluoride membranes. After blocking, membranes were incubated overnight at 4 °C with primary antibodies against CD24 or glyceraldehyde-3-phosphate dehydrogenase. Horseradish peroxidase-conjugated secondary antibodies were used for detection, and chemiluminescent signals were recorded using commercial enhanced chemiluminescence reagents. Band intensities were quantified using ImageJ.

### Quantitative real-time PCR

Total RNA was isolated using TRIzol reagent, treated with DNase I, and reverse-transcribed using a SuperScript synthesis kit. Quantitative real-time polymerase chain reaction (qRT-PCR) was performed with SYBR-based detection chemistry. Gene expression levels were normalized to 18S ribosomal RNA, and relative quantification was calculated using the 2^−ΔΔCt^ method. Primers are listed in Table S2.

### Dual-luciferase reporter gene assay

Predicted binding interactions between miR-172f-p3 and the CD24 3′ untranslated region (UTR) were obtained using StarBase. Wild-type and mutant CD24 3′ UTR segments were cloned into psiCHECK-2 vectors. HEK293 cells were cotransfected with these constructs together with miRNA mimics or negative controls (NCs). Luminescence was measured using a dual-luciferase assay system.

### RNA library construction and miRNA sequencing

Total RNA from light-treated and untreated HPDENs (3 replicates per group) was extracted using TRIzol. RNA concentration and quality were assessed by NanoDrop and Bioanalyzer. Small-RNA library preparation and sequencing were conducted by a commercial provider following standard protocols. Data normalization, differential expression analysis, and functional annotation (Gene Ontology [GO] and Kyoto Encyclopedia of Genes and Genomes [KEGG]) were performed using established bioinformatic pipelines.

### Bioinformatic analysis

The Feature Extraction software (version 10.7.1.1, Agilent Technologies) was used to analyze array images to get raw data. Next, the raw data were normalized with the quantile algorithm. The probes detected with at least 75.0% in any group were chosen for further data analysis. Differentially expressed miRNAs were then identified through fold change as well as the *P* value calculated using a *t* test. The threshold set for up- and down-regulated genes was a fold change ≥2.0 and a *P* value ≤0.05. The target genes of differentially expressed miRNAs were the intersection predicted with 2 databases (miRDB and miRWalk). GO analysis and KEGG analysis were applied to determine the roles of these target genes. Hierarchical clustering was performed to show the distinguishable miRNA expression pattern among samples.

### Cellular uptake of HPDENs

MCF-7 cells were incubated with fluorescently labeled HPDENs under light-treated or untreated conditions. Nuclei were counterstained with 4′,6-diamidino-2-phenylindole prior to imaging. Internalization of vesicles was visualized using fluorescence microscopy, and uptake patterns were compared across time points.

### miRNA mimic transfection and efficiency evaluation

To assess the functional role of miR-172f-p3, miRNA mimic transfection was performed in MCF-7 cells using the riboFECT CP Transfection Kit (Guangzhou Ribo Biologics) according to the manufacturer’s protocol. Cells were transfected with an miR-172f mimic or the NC at a final concentration of 100 nmol/l for 24 h.

Transfection efficiency was evaluated indirectly by measuring intracellular miRNA levels and target gene modulation. The relative expression of miR-172f-p3 was quantified by qRT-PCR, and the expression of its target gene CD24 was assessed at the protein level using Western blot analysis. miRNA sequences are provided in Table S2.

### Flow cytometric analysis

Fluorescence-activated cell sorting (FACS) was performed as previously described [18]. RAW264.7 cells were preincubated with anti-CD16/32 for Fc-block. Cells were then stained with anti-Siglec-G/10 monoclonal antibodies on ice. Signals were recorded using a FACS Canto II instrument, and data are expressed as percentages of positive cell subsets.

### In vivo tumor model and treatment protocol

MCF-7 cells were preincubated with photoactivated HPDENs (15 μg/ml) for 24 h prior to subcutaneous implantation into mice. No additional HPDEN administration was performed in vivo. Female BALB/c-nude mice (6 to 8 weeks old) were purchased from Chongqing Tengxin Bio-Tech Co., Ltd. Mice were housed in pathogen-free cages (5 animals per cage) with a 12-h light/dark cycle, controlled temperature (22 ± 2 °C), and humidity (55% ± 10%). MCF-7 cells (5 × 10^7^ cells per site) pre-treated in vitro for 24 h with light-activated HPDENs, non-light-treated HPDENs, miR-172f-p3 mimic, or appropriate controls were injected orthotopically into the third/fourth mammary fat pads (one site per mouse). Tumor volumes were measured every 2 to 3 d using calipers and calculated as (length × width^2^)/2. Tumor size and body weight were measured and recorded every 5 d for a total of 15 d. All procedures were performed in accordance with the Institutional Animal Care and Use Committee of Southwest Medical University.

### Histological and immunohistochemistry

Tumor samples were fixed in 4% paraformaldehyde, cryoprotected in sucrose, embedded, and sectioned. For immunohistochemical detection, sections were incubated with anti-CD24 antibodies, followed by biotin-labeled secondary antibodies. Stained sections were imaged using a digital microscope, and CD24-positive areas were quantified using image analysis software.

### Statistical analysis

Data are expressed as mean ± standard error of the mean. Comparisons between groups were performed using one-way analysis of variance followed by appropriate post hoc tests. Statistical significance was set at *P* < 0.05.

## Results

### Photoactivation of HPDENs modulates miRNA cargo and specifically up-regulates miR-172f-p3, targeting CD24 expression

Following isolation, HPDENs were characterized to confirm their properties. NTA revealed a mean particle size of 125 ± 2.15 nm for light-treated and 168 ± 3.22 nm for non-light-treated HPDENs. Zeta potentials were −15.2 ± 0.01 and −16.6 ± 0.05 mV, respectively. These results provide baseline physical metrics for the vesicles used in subsequent experiments.

**Fig. 1.**
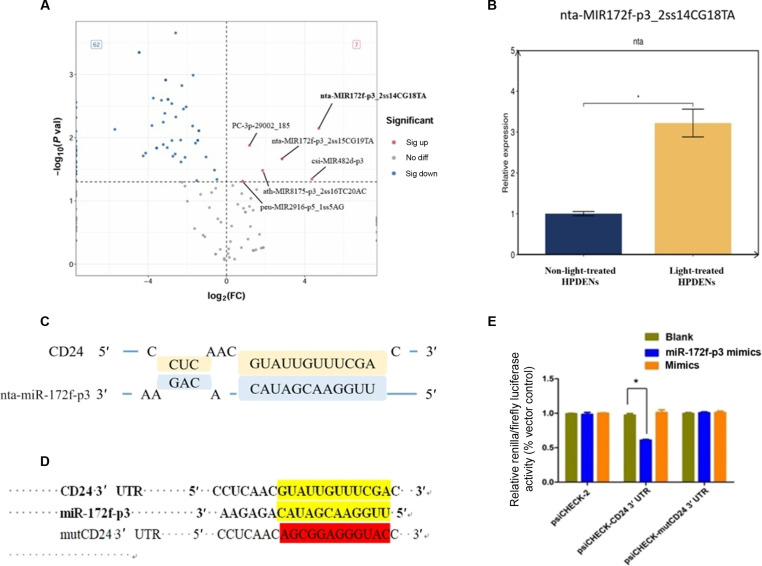
Photoactivated up-regulation of miR-172f in exosome-like nanovesicles and its targeting of the CD24 gene. (A) Volcano plot analysis of differentially expressed microRNAs (miRNAs) in light-treated versus non-light-treated exosome samples. (B) Quantitative real-time polymerase chain reaction (qRT-PCR) validation of miR-172f-p3 expression in light-treated and non-light-treated exosomes (*n* = 3, *P < 0.05). (C) Schematic representation of the bioinformatics prediction for the binding site between the CD24 3′ untranslated region (UTR) sequence and miR-172f-p3 (database: National Center for Biotechnology Information; RefSeq: NM_001291737.1; prediction tool: RNAhybrid v2.2). (D and E) Construction of the dual-luciferase reporter system and validation of the targeting interaction between miR-172f-p3 and CD24 (*n* = 3, *P < 0.05). HPDENs, *Hypericum perforatum*-derived exosome-like nanovesicles.

Our prior miRNA sequencing analysis of HPDENs revealed that light exposure induced differential expression of 82 miRNA types, with 11 up-regulated and 71 down-regulated [9]. Among these, miR-172f-p3 was markedly up-regulated following photoactivation. We subsequently validated this increase in nta-miR-172f-p3 expression using qPCR. However, the mechanisms underlying miRNA differential expression mediated by light-activated medicinal-plant-derived exosomes, as well as their role in the cross-kingdom regulation of human diseases, remain to be fully elucidated. First, we performed a systematic analysis of the miRNA expression profiles. Compared with non-light-treated HPDENs, light exposure triggered a reprogramming of the miRNA landscape and identified multiple significantly up-regulated miRNAs (*P* < 0.05, log_2_FC > 0), as illustrated in (Fig. 1A). Notably, the expression of *nta-miR-172f*, derived from *H. perforatum* (St. John’s wort) exosomes, increased significantly following light irradiation. This suggests that *nta-miR-172f* may function as a light-responsive miRNA involved in HPDEN-mediated functional regulation. Subsequently, we validated the up-regulation of *nta-miR-172f* in the light-treated HPDEN group using qPCR assays (Fig. 1B).

To further reveal its potential targets, we conducted bioinformatics prediction and screening supplemented by literature review [6]. Our analysis identified a highly complementary binding sequence between *nta-miR-172f* and the 3′ UTR of human *CD24* messenger RNA (mRNA); the pairing between the miRNA and its target sequence is shown in (Fig. 1C). Furthermore, dual-luciferase reporter assays confirmed that *nta-miR-172f* specifically binds to the *CD24* 3′ UTR and significantly suppresses reporter activity. This inhibitory effect was abolished when the binding site was mutated, thereby verifying *CD24* as a direct functional target of *nta-miR-172f* (Fig. 1D and E).

In summary, light activation not only triggers extensive reprogramming of the miRNA cargo within HPDENs but also specifically up-regulates *nta-miR-172f*, which possesses the potential to target *CD24*. This indicates that light may endow HPDENs with gene regulatory functions that extend beyond traditional photodynamic effects by modulating the exosomal miRNA profile, providing a theoretical foundation for their subsequent roles in tumor cell *CD24* inhibition and immune microenvironment reprogramming.

### Cellular uptake and CD24 modulation by photoactivated HPDENs

**Fig. 2.**
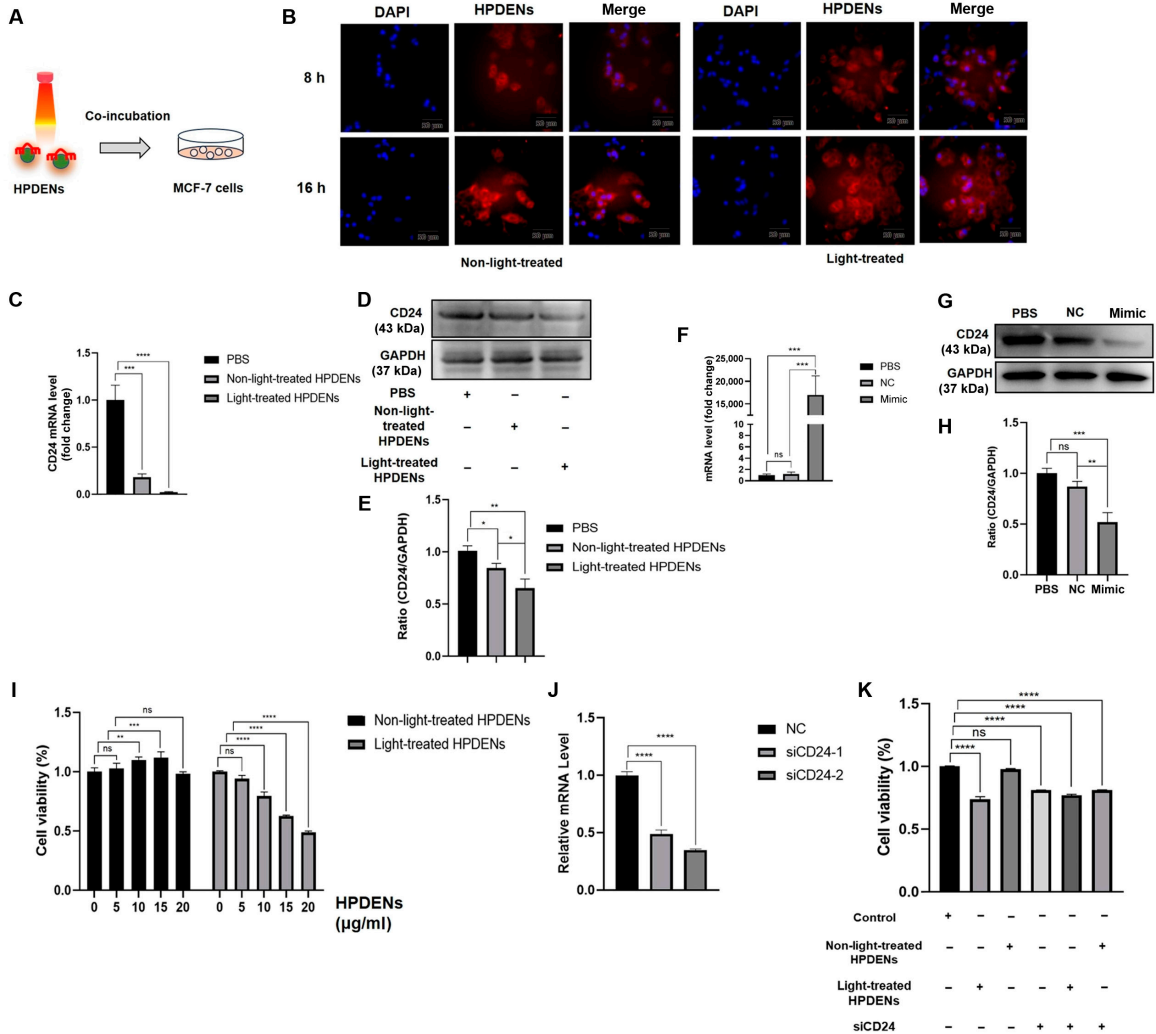
Uptake and CD24 modulation by photoactivated *Hypericum perforatum*-derived exosome-like nanovesicles (HPDENs) in MCF-7 cells. (A and B) Fluorescent examination of the cellular uptake of HPDENs (0.1 g/l) in MCF-7 cells at the indicated time of incubation, showing synchronous localization of HPDENs with both the cytoplasm and extracellular vesicles. Scale bar, 50 μm. Experiments were repeated independently for 3 times. (C) Quantitative real-time polymerase chain reaction (qRT-PCR) analysis of CD24 expression in MCF-7 cells. (D and E) Western blot analysis of CD24 expression in MCF-7 cells treated with HPDENs. The band intensity is assessed. (F) Functional delivery of miR-172f-p3 mimics in MCF-7 cells. qRT-PCR analysis shows fold change of intracellular miR-172f-p3 levels after transfection with a microRNA (miRNA) mimic or the negative control (NC). CD24 expression was assessed by Western blot. Data are shown as mean ± standard error of the mean (SEM); ***P < 0.001; ns, not significant (*n* = 4). (G and H) Western blot analysis of CD24 expression in MCF-7 cells transfected with MIMIC-miR-172f-p3. The band intensity is assessed. All graphs correspond to the blots above them and represent densitometric analyses of 3 independent experiments. **P < 0.01. (I) Cells were co-cultured with different concentrations of HPDENs for 24 h to measure cell viability using a Cell Counting Kit-8 (CCK-8) assay. The phosphate-buffered saline (PBS) group served as NC. Data are expressed as means ± SEM. (J and K) Cells were transfected with CD24 small interfering RNA (siRNA). qRT-PCR analysis of CD24 expression in MCF-7 cells. Cells treated with HPDENs for 24 h were subjected to assess cell viability. *P < 0.05; **P < 0.01; ***P < 0.001.

MCF-7 cells were selected for their high CD24 expression, a key immune checkpoint in breast cancer. Following the experimental design depicted in Fig. 2A, we incubated photoactivated HPDENs (after 30 min of light exposure) with MCF-7 breast cancer cells. We then investigated the cellular internalization of HPDENs using both confocal microscopy. Images captured after 8 and 12 h of co-incubation with MCF-7 breast cancer cells showed a gradual increase in fluorescence intensity over time, indicating progressive cellular uptake of the HPDENs (Fig. 2B).

Next, we evaluated the impact of HPDENs on CD24 expression in MCF-7 cells using both qPCR and immunoblotting. Compared to the phosphate-buffered saline (PBS) control group, both light-treated and non-light-treated HPDENs significantly inhibited CD24 expression in MCF-7 cells. Notably, the inhibitory effect on CD24 expression was more pronounced with light-treated HPDENs than with non-light-treated HPDENs (Fig. 2C to E). To evaluate the functional role of miR-172f-p3, miRNA mimics or NC was directly transfected into MCF-7 cells (100 nmol/l, 24 h). qRT-PCR analysis showed robust up-regulation of miR-172f-p3 in mimic-treated cells compared to PBS or NC, accompanied by significant down-regulation of the target gene CD24 (Fig. 2F). Similar results were obtained when breast cancer cells were transfected with a synthetic mimic of miR-172 (Fig. 2G and H).

Regarding cell viability, light-treated HPDENs demonstrated a significant and concentration-dependent inhibitory effect. In contrast, non-light-treated HPDENs did not exhibit a noticeable inhibitory effect on cell viability (Fig. 2I). To further confirm that this reduction in cell viability was mediated through CD24, we screened and selected effective CD24-targeting small interfering RNAs (siRNAs). Subsequently, MCF-7 cells were either treated with light-treated HPDENs, treated with non-light-treated HPDENs, or transfected with CD24 siRNA. The results demonstrated a significant reduction in breast cancer cell viability in both the light-treated HPDEN group and the CD24 knockdown group (Fig. 2J and K). This parallelism strongly suggests that miRNA-mediated down-regulation of CD24 by light-treated HPDENs significantly contributes to their antiproliferative effect, indicating a synergistic therapeutic mechanism.

### Photoactivated HPDENs reprogram macrophage polarization via miR-172f-p3-mediated CD24 down-regulation

**Fig. 3.**
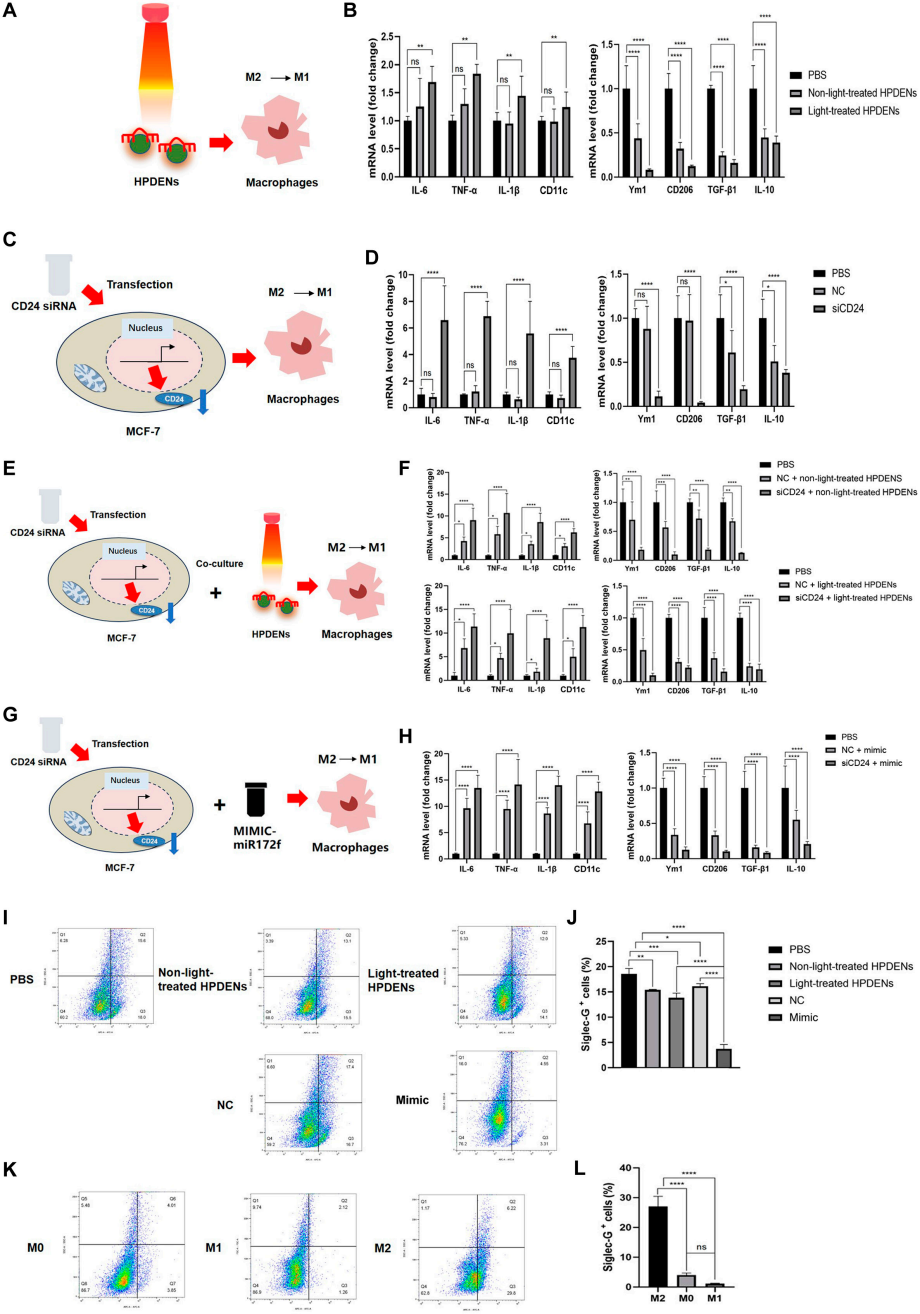
Photoactivated *Hypericum perforatum*-derived exosome-like nanovesicles (HPDENs) reprogram macrophage polarization via miR-172f-p3-mediated CD24 down-regulation. (A and B) MCF-7 cells with either light-treated or non-light-treated HPDENs. Subsequently, these treated MCF-7 cells were co-incubated with pre-induced M2-polarized macrophages. Quantitative real-time polymerase chain reaction (qRT-PCR) analysis was performed to assess the expression of both M1 and M2 macrophage markers. (C and D) Co-incubation experiments were conducted following CD24 small interfering RNA (siRNA) transfection in MCF-7 cells. qRT-PCR analysis was performed to assess the expression of both M1 and M2 macrophage markers. NC, negative control. Cells were silenced with CD24 siRNA and treated with either light-treated or non-light-treated HPDENs (E and F) or transfected with MIMIC-miR-172f-p3 (G and H). The MCF-7 cells were then co-incubated with pre-induced M2 macrophages, and M1/M2 markers were assessed by qRT-PCR. (I and J) Siglec-G/10 expression was evaluated by flow cytometry on the surface of macrophages under the indicated treatment conditions. (K and L) Siglec-G/10 expression was assessed on M0–M1–M2-polarized macrophages, respectively. The data represent the mean of 3 independent experiments. *P < 0.05; **P < 0.01; ***P < 0.001; ****P < 0.0001.

To investigate whether the observed reduction in CD24 expression leads to a shift in macrophage polarization, we first treated MCF-7 cells with either light-treated or non-light-treated HPDENs. Subsequently, these treated MCF-7 cells were co-incubated with pre-induced M2-polarized macrophages. We then assessed the expression of both M1 and M2 macrophage markers by qRT-PCR. As shown in Fig. 3A and B, the mRNA levels of pro-inflammatory genes, including IL-6, IL-1β, TNF-α, and CD11c, were significantly up-regulated in the light-treated HPDENs, compared with those in non-light-treated HPDEN and PBS groups. Similarly, the light-treated HPDENs down-regulated the mRNA levels of IL-10, YM1, TNF-β, and CD206.

Given that CD24 is expressed on MCF-7 cells, we investigated whether alterations in CD24 directly influence M1–M2 polarization. Co-incubation experiments were conducted following CD24 siRNA transfection in MCF-7 cells. The results demonstrated a significant up-regulation of M1 markers in the CD24 siRNA group compared to those in the NC group, with an approximately 4-fold increase. Concurrently, the CD24 siRNA group exhibited a notable down-regulation of M2 markers, including the mRNA levels of IL-10, YM1, TNF-β, and CD206 (Fig. 3C and D). These findings suggest that the knockdown of CD24 induce a shift in macrophage polarization from an M2 to an M1 phenotype through the regulation of CD24 on MCF-7 cells.

To precisely confirm the role of light-treated HPDENs and miR-172f-p3 in mediating the CD24-dependent macrophage polarization shift, we conducted experiments where MCF-7 cells, after CD24 knockdown, were either treated with light-treated HPDENS, treated with non-light-treated HPDENs, or transfected with an miR-172f-p3 mimic. The MCF-7 cells were then co-incubated with pre-induced M2 macrophages, and M1/M2 markers were assessed by qRT-PCR (Fig. 3E to H). The results showed that transfection with miR-172f-p3 significantly enhanced the CD24 knockdown-mediated macrophage polarization shift.

To further understand the involvement of the CD24–Siglec-G/10 axis in the light-treated HPDENs and miR-172f-p3-mediated macrophage polarization shift, we analyzed Siglec-G/10 expression on the surface of macrophages using flow cytometry under various treatment conditions. Our findings revealed that in the context of macrophage polarization shifts mediated by the decreased CD24 expression due to light-treated HPDENs and miR-172f-p3, Siglec-G/10 expression on M1-polarized macrophages was significantly lower than that on M2-polarized macrophages (Fig. 3I to L). These comprehensive results collectively indicate that light-treated HPDENs, through miR-172f-p3 cargo and subsequent down-regulation of CD24, actively contribute to reprogramming the immunosuppressive tumor microenvironment by promoting M1 macrophage polarization via the CD24–Siglec-G/10 signaling pathway. Siglec-G (in mice) and Siglec-10 (in humans) are inhibitory receptors that typically bind to CD24 on cancer cells, delivering “don’t eat me” signals that suppress macrophage phagocytic activity and pro-inflammatory responses. Therefore, the low expression of Siglec-G/10 on M1-polarized macrophages indicates a reduction in inhibitory signals received from CD24, allowing them to more effectively exert their antitumor functions [15–17]. This also helps M1 macrophages maintain their pro-inflammatory and antitumor characteristics, as they are no longer inhibited by the CD24–Siglec-G/10 axis. Conversely, the high expression of Siglec-G/10 on M2-polarized macrophages likely forms stable binding with highly expressed CD24, maintaining their immunosuppressive characteristics and helping tumors evade immune surveillance.

Collectively, the observed direct effect of light-treated HPDENs on M1 polarization was notably less pronounced than the significant up-regulation of M1 marker genes achieved when light-treated HPDENs modulated CD24 expression on MCF-7 cells, indicating that the immunomodulatory effects of light-treated HPDENs are primarily mediated through alterations in tumor cell CD24.

### In vivo tumor therapy of photoactivated HPDENs

**Fig. 4.**
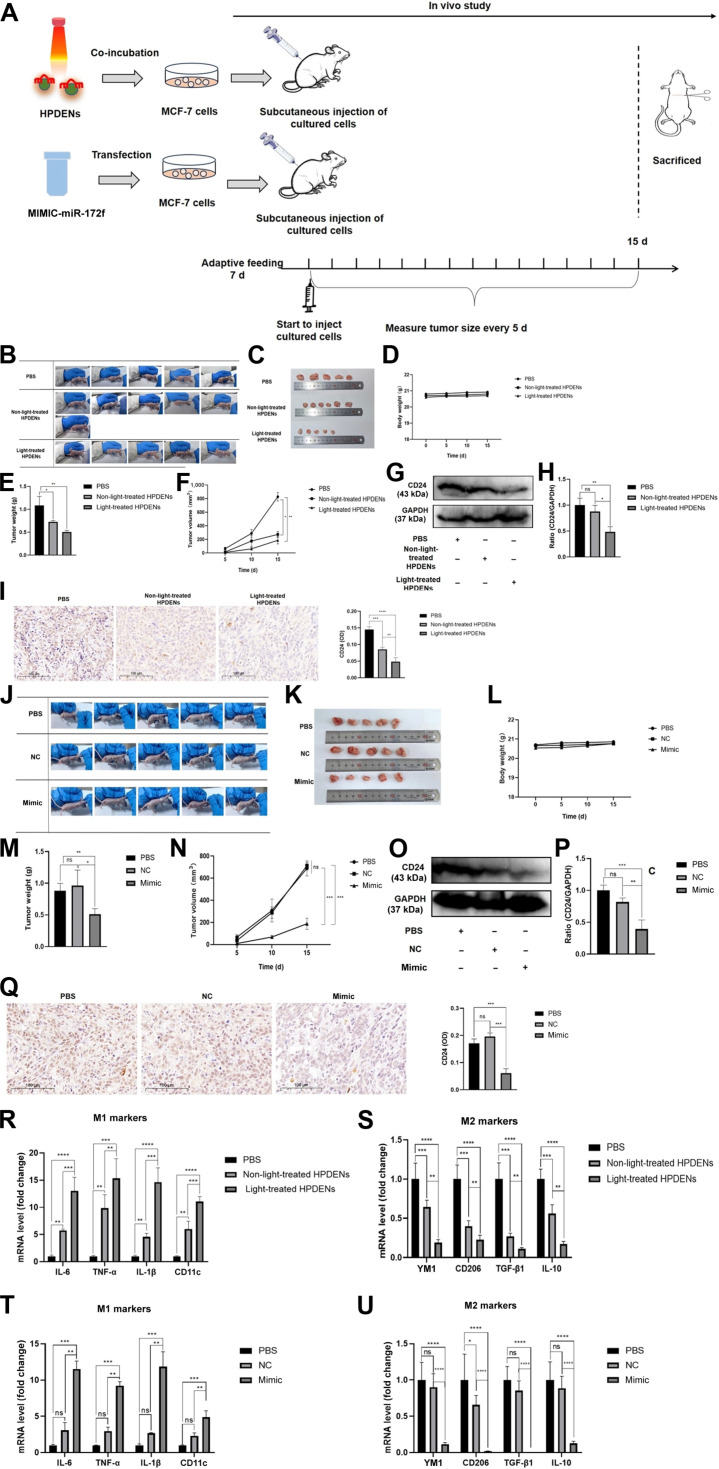
In vivo tumor therapy of photoactivated *Hypericum perforatum*-derived exosome-like nanovesicles (HPDENs). (A) Schematic timeline of the in vivo tumor model of light-treated HPDENs. Co-culture of light-treated HPDENs with MCF-7 cells for 24 h in vitro; these cells were then implanted into nude mice to establish a tumor growth model, dividing the mice into an HPDEN-treated group and a MIMIC-miR-172f-p3-treated group. Observation continued until day 15; the body weight and tumor size of the nude mice were recorded. (B and C) Photographs of tumors generated from phosphate-buffered saline (PBS), light-treated HPDEN, and non-light-treated HPDEN nude mice (*n* = 5 to 6/each group). (D to F) Body and tumor weights and tumor volume were measured 15 d after cell injection. (G and H) Western blot analysis of CD24 expression in tumor tissues as indicated. The band intensity was calculated. (I) Representative images of immunohistochemistry analysis of CD24-positive cells in paraffin sections from tumors. Quantification of the immunostaining intensity of each group. Scale bar, 100 μm. (J and K) Photographs of tumors generated from PBS, negative control (NC), and MIMIC-miR-172f-p3-treated nude mice (*n* = 5/each group). (L to N) Body and tumor weights and tumor volume were measured 15 d after cell injection. (O and P) Western blot analysis of CD24 expression in tumor tissues as indicated. The band intensity was calculated. (Q) Representative images of immunohistochemistry analysis of CD24-positive cells in paraffin sections from tumors. Quantification of the immunostaining intensity of each group. Scale bar, 100 μm. (R and S) Total RNA was extracted from tumor tissues generated from PBS, light-treated HPDEN, and non-light-treated HPDEN nude mice. Quantitative real-time polymerase chain reaction (qRT-PCR) analysis was performed to assess the expression of both M1 and M2 macrophage markers. (T and U) Total RNA was extracted from tumor tissues generated from PBS, NC, and MIMIC-miR-172f-p3-treated nude mice nude mice. qRT-PCR analysis was performed to assess the expression of both M1 and M2 macrophage markers. The data represent the mean of 3 independent experiments. *P < 0.05; **P < 0.01; ***P < 0.001; ****P < 0.0001.

To clarify the in vivo antitumor potential of light-treated HPDENs, we co-incubated light-treated HPDENs with cultured MCF-7 cells for 24 h in vitro, as shown in Fig. 4A. These cells were then implanted into nude mice to establish a tumor growth model, dividing the mice into a light-treated group and a non-light-treated HPDEN group. Observation continued until day 15, during which we carefully recorded the body weight and tumor size of the nude mice. After 15 d, a noticeable and significant tumor shrinkage was observed in the light-treated HPDEN group, whereas no significant changes were observed in the non-light-treated group and the PBS group (Fig. 4B). Body weight remained stable within the 15 d (Fig. 4C). Notably, the growth of tumor tissue in the light-treated HPDEN group was significantly inhibited, with its volume significantly smaller than that in the non-light-treated group and the PBS group (Fig. 4D and E). In parallel mimic animal experiments, treatment also continued until day 15, during which we observed and recorded the nude mice’s body weight and tumor size (Fig. 4I to K). Similar to the light-treated HPDEN group, comparing the NC control group and the PBS group, the miR-172f-p3 mimic group showed significant inhibition of tumor tissue growth (Fig. 4L and M). In addition, we utilized immunoblotting and immunohistochemistry to examine changes in CD24, and the results showed that both the light-treated HPDENs (Fig. 4G to I) and the miR-172f-p3 mimic group (Fig. 4O to Q) displayed significant suppression of CD24 expression in the tumor tissues. These results highlight the potent in vivo antitumor efficacy of both light-treated HPDENs and the miR-172f-p3 mimic.

To evaluate macrophage polarization in the tumor microenvironment, we assessed the expression of both M1 and M2 macrophage markers in the tumor tissues using qRT-PCR. As depicted in Fig. 4R and S, treatment with light-treated HPDENs drove a significant shift toward a pro-inflammatory M1 phenotype. This was evidenced by the robust up-regulation of M1-associated genes, including IL-6, IL-1β, TNF-α, and CD11c, and the concurrent down-regulation of M2-associated genes, such as IL-10, YM1, TNF-β, and CD206, when compared to those in the non-light-treated and PBS control groups. This M1/M2 gene expression profile was similarly replicated in the mimic miR-172 group (Fig. 4T and U), confirming its parallel role in modulating macrophage polarization.

### Biocompatibility

Our prior in vivo investigation established the favorable biocompatibility of HPDENs following PDT [9,17]. To further evaluate the systemic safety of light-treated HPDENs and the miR-172f-p3 mimic in vivo, we performed a comprehensive serum analysis, assessing liver, gallbladder, hepatobiliary, and kidney functions (Fig. S1A and B), along with a complete blood count (Table S1A and B). Results indicated no abnormalities in functional parameters for either the light-treated HPDENs or miR-172f-p3 mimic groups, with all values remaining within normal physiological ranges. Conversely, the PBS control group exhibited significant elevations in white blood cells, lymphocytes, monocytes, and granulocytes, as determined by complete blood count. Both light-treated and non-light-treated HPDENs maintained all hematological parameters within normal limits. Consistent with these findings, the miR-172f-p3 mimic group also demonstrated normal hematological values, in contrast to the elevated levels observed in the PBS and NC groups. These results collectively confirm the biocompatibility of both light-treated HPDENs and the miR-172f-p3 mimic following local administration.

## Discussion

This study reveals a previously unrecognized mechanism by which photoactivated HPDENs potentiate antitumor immunity, extending their function beyond classical PDT [9,11]. While conventional PDT primarily induces cytotoxicity via ROS generation, our findings demonstrate that HPDENs possess a dual-mode therapeutic capacity: direct tumor suppression mediated by miRNA-dependent CD24 down-regulation and indirect remodeling of the tumor immune microenvironment through inhibition of the CD24–Siglec-G/10 phagocytosis checkpoint [10–14].

Photoactivation-induced miRNA reprogramming in HPDENs may be associated with a localized redox microenvironment generated during light exposure. Based on our previous findings [9], 590-nm irradiation of HPDENs induces significant intracellular ROS production without disrupting vesicle integrity, suggesting a controlled photoredox process rather than nonspecific structural damage. We propose that this ROS burst may modulate redox-sensitive RNA-binding proteins (RBPs) packaged within HPDENs, such as hnRNPA2B1, SYNCRIP, and FMR1, which are known to regulate selective miRNA sorting in extracellular vesicles [15–17]. Oxidative modifications of these RBPs may alter their conformational state and RNA-binding affinity, thereby influencing the selective stabilization and retention of specific miRNAs. In this context, miR-172f-p3 may exhibit enhanced stability or preferential association with RBPs, resulting in its relative enrichment following photoactivation. This hypothesis is consistent with previous reports demonstrating that oxidative stress can regulate extracellular vesicle miRNA cargo composition through RBP-dependent mechanisms. Therefore, the observed increase in miR-172f-p3 is unlikely to result from vesicle disruption but rather reflects a regulated, ROS-associated miRNA sorting process. Further proteomic analysis and RNA immunoprecipitation-based approaches will be required to identify the specific RBPs involved and validate this mechanism.

CD24 is a crucial innate immune checkpoint that interacts with Siglec-10/G on macrophages to deliver inhibitory “don’t-eat-me” signals [11–14]. By suppressing CD24 expression, photoactivated HPDENs disrupt this ligand–receptor interaction and promote macrophage activation. This establishes a mechanistic link between exosomal miRNA modulation and innate immune reprogramming [18–20].

Importantly, our data show that HPDEN-mediated immunomodulation occurs primarily through tumor-cell-dependent mechanisms rather than direct macrophage stimulation. Photoactivated HPDENs alone induced limited macrophage activation, whereas their effects were significantly amplified when tumor cells served as intermediaries that relayed molecular alterations—particularly CD24 suppression—to macrophages. This aligns with growing evidence that tumor-cell-centered immunomodulation is an effective means to reshape the tumor microenvironment [21–23]. Based on our previous study [9], HPDENs show a clear irradiation-time-dependent photodynamic response under 590-nm light, with ROS levels (including singlet oxygen, superoxide anions, and hydroxyl radicals) increasing with exposure time. This provides a photokinetic basis for HPDEN activation. Since ROS is upstream of miR-172f-p3 up-regulation and CD24 suppression, irradiation duration is likely to influence CD24 modulation. In this study, a fixed irradiation condition was used for consistency across experiments, focusing on the HPDEN–miR-172f-p3–CD24 axis. CD24 expression under different irradiation times was not systematically evaluated and will be addressed in future work to define the optimal photodynamic window.

In vivo experiments further validated these findings: both photoactivated HPDENs and miR-172f-p3 mimic treatment inhibited tumor growth, suppressed CD24 expression, and promoted a robust M1 macrophage phenotype. The absence of hepatic, renal, or hematologic abnormalities supports the favorable biosafety profile of HPDEN-based therapeutics [20,24].

This study demonstrates the dual antitumor and immunomodulatory effects of photoactivated HPDENs, but several limitations should be acknowledged. Experiments were mainly conducted using the MCF-7 luminal breast cancer model, which may limit generalizability, and future studies should include additional subtypes such as HER2-positive and triple-negative models. In vivo studies used nude mice, allowing assessment of macrophage polarization but not adaptive immunity, including T-cell responses and immune memory, which should be addressed in immunocompetent or humanized models. The observed antitumor effects involve both ROS generation and miR-172f-p3-mediated CD24 suppression, but the relative contributions were not separated using ROS inhibitors or miRNA knockdown. Pharmacokinetics, biodistribution, clearance, and long-term toxicity were not assessed, as the in vivo model relied on pre-treated tumor cells rather than systemic HPDEN administration. Tumor-targeting selectivity and organ distribution were also not evaluated. Multiple batches of HPDENs were consistent in size, morphology, and concentration at the laboratory scale, but large-scale production and standardized quality control under good manufacturing practice (GMP) conditions remain to be established. While miR-172f-p3 was studied as a key mediator, HPDENs contain a diverse repertoire of other differentially expressed miRNAs that may exert coordinated or synergistic effects; functional validation of these miRNAs is needed. Optimization of nanoparticle engineering—including miRNA loading, light irradiation/photoactivation parameters, and tumor-targeting features—may further enhance HPDEN photoactivation efficiency, downstream CD24 modulation, and overall therapeutic performance. The in vivo experiments employed a cell-priming strategy, and no dose–response studies were performed; future work will focus on optimizing systemic delivery and dosing regimens to evaluate therapeutic efficacy. Collectively, these limitations highlight areas for further research to refine and fully understand the mechanistic and translational potential of photoactivated HPDENs.

**Fig. 5.**
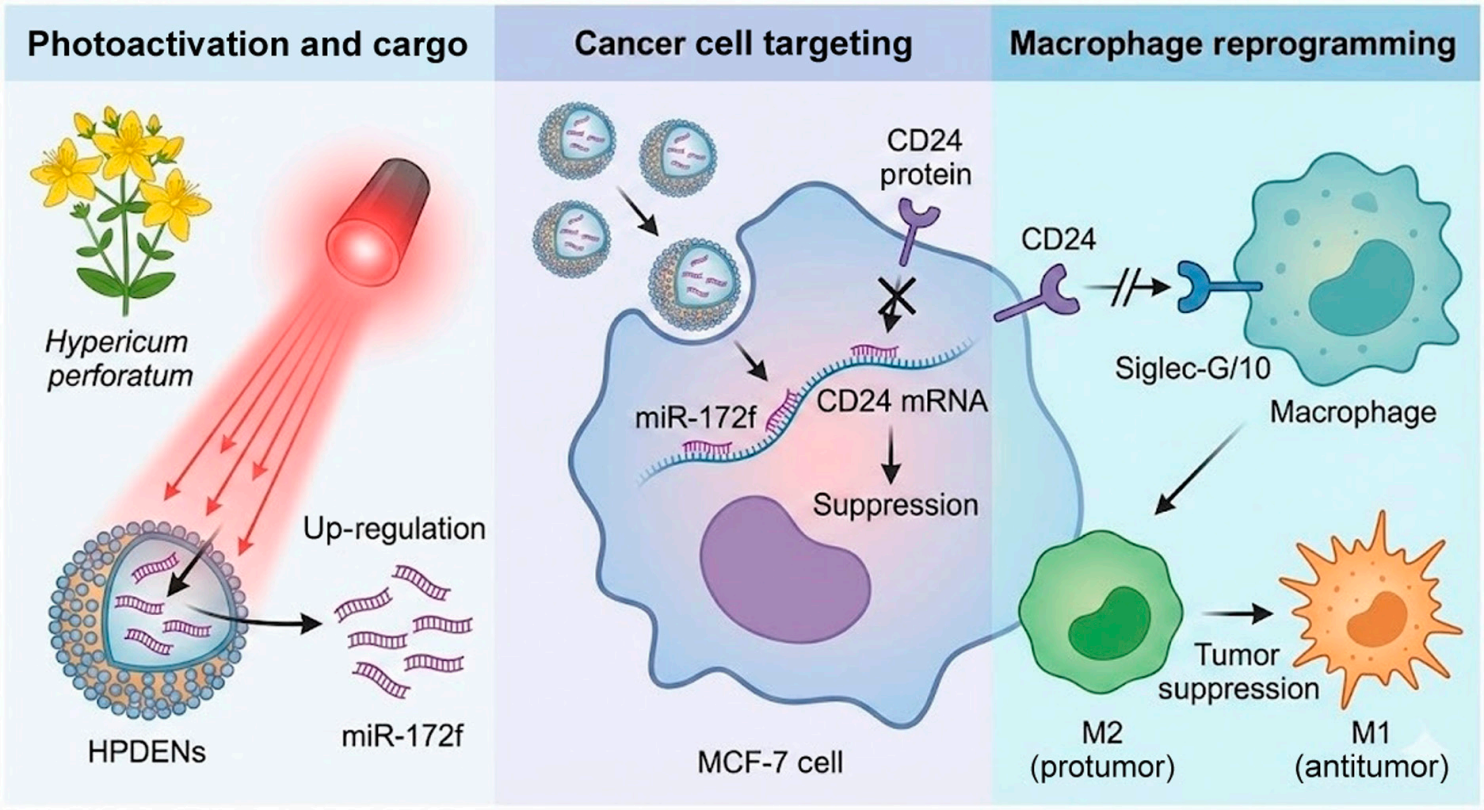
Photoactivated *Hypericum perforatum*-derived exosome-like nanovesicles (HPDENs) alter M2-to-M1 conversion by reducing CD24 expression in MCF-7 cells via the miR-172f-p3/CD24–Siglec-G/10 pathway. Photoactivation of *H. perforatum*-derived nanovesicles (HPDENs) selectively enriches miR-172f-p3 cargo. Upon internalization by breast cancer cells, miR-172f-p3 targets the 3′ untranslated region (UTR) of CD24 messenger RNA (mRNA), silencing CD24 expression. This disruption of the CD24–Siglec-10/G “don’t eat me” axis reprograms tumor-associated macrophages from an M2-like state to a pro-inflammatory M1 phenotype, restoring antitumor phagocytosis.

Overall, our findings establish photoactivated HPDENs as a multifunctional nanoplatform integrating photodynamic activity with miRNA-mediated immune checkpoint regulation. By modulating the miR-172f-p3/CD24–Siglec-G/10 axis, HPDENs effectively reprogram macrophage phenotypes and suppress tumor progression, providing a strong rationale for future clinical development of plant-derived exosomal nanovesicles as next-generation photodynamic–immunoregulatory therapeutics (Fig. 5). The reproducibility and scalability of HPDEN production are essential for clinical translation. In this study, HPDENs were isolated using a standardized protocol involving differential centrifugation and ultrafiltration and characterized by NTA, transmission electron microscopy, and protein content analysis. Multiple independent batches demonstrated consistent vesicle size, concentration, and morphology, confirming laboratory-scale reproducibility. For clinical translation, scalable production strategies such as tangential flow filtration, standardized plant biomass processing, and rigorous quality control measures will be required to ensure batch-to-batch consistency and compliance with GMP standards. These considerations provide a framework for the translation of HPDEN-based therapeutics from bench to clinic.

## Ethical Approval

All animal procedures were approved by the Experimental Animal Ethics Committee of Southwest Medical University (20250227-004).

## Data Availability

Data are provided within the article or Supplementary Materials file.
