# Supplementary material for: Photoactivated Hypericum perforatum-Derived Exosome Nanovesicles Suppress Breast Cancer via miR-172f/CD24–Siglec-G/10-Driven Macrophage Reprogramming
Source: Biomater Res. 2026 Jul 17;30:0393. doi: 10.34133/bmr.0393 (PMC13376378; doi:10.34133/bmr.0393)
Supplement: Supplementary 1 — Fig. S1 Tables S1 and S2 [file bmr.0393.f1.pdf]

Supplemental Figure 1

A

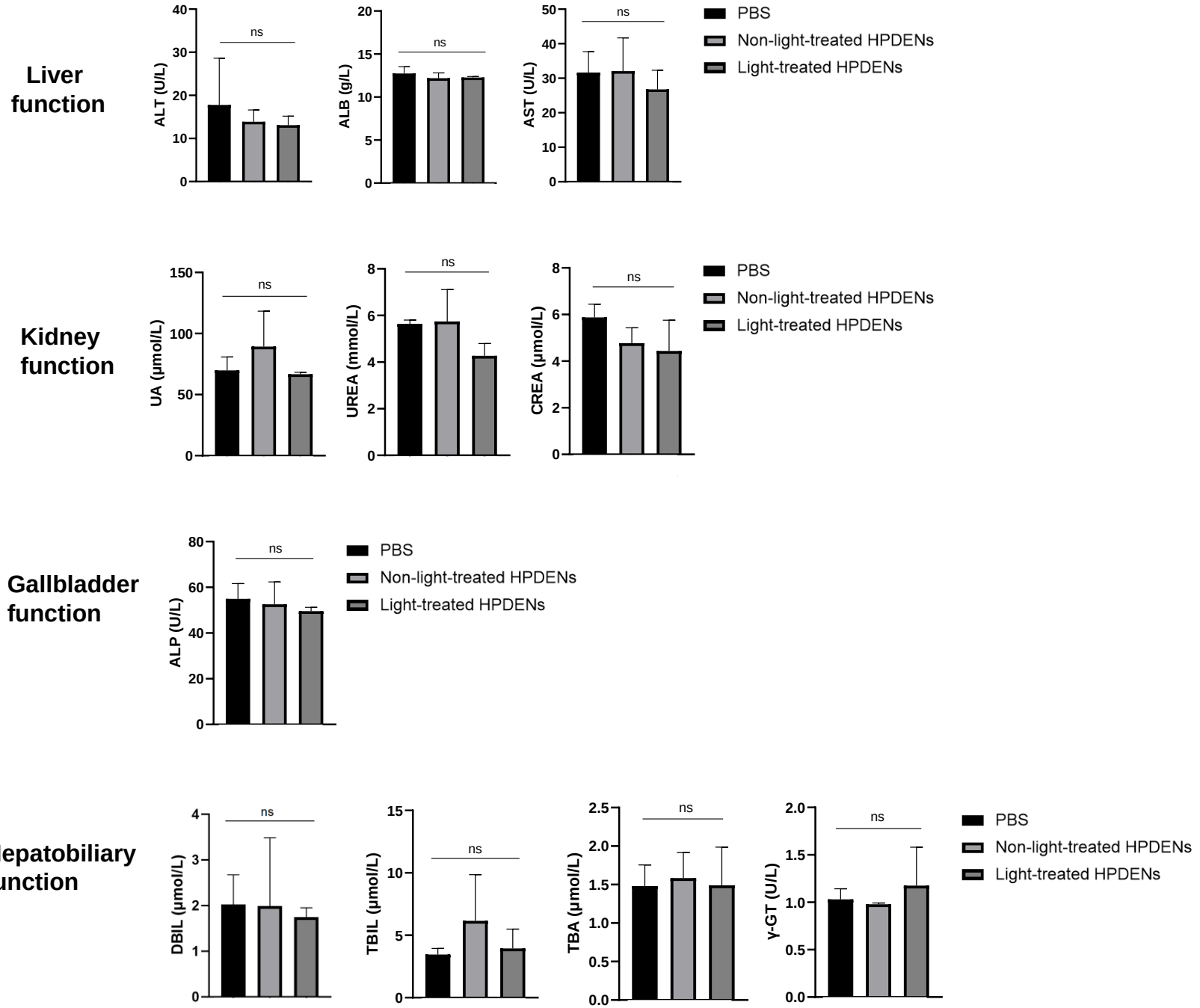

B

Biocompatibility of NC or MIMIC transfection treatments

Liver function

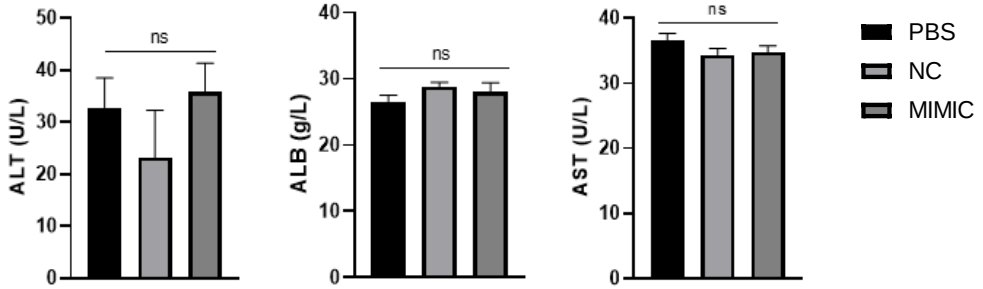

Kidney function

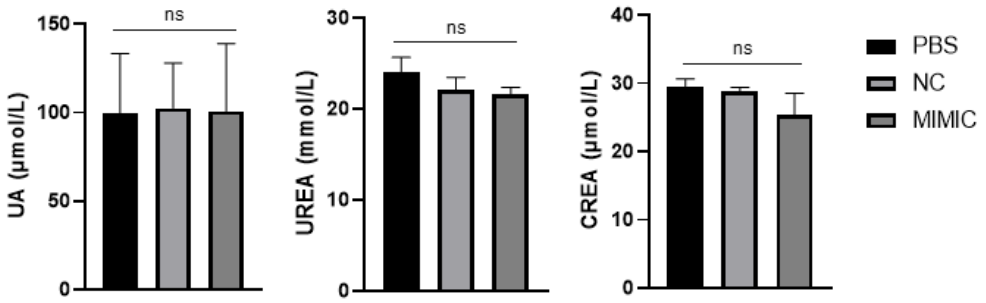

Gallbladder function

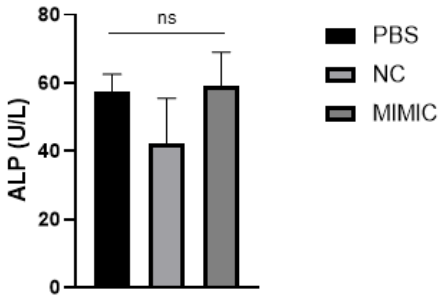

Hepatobiliary function

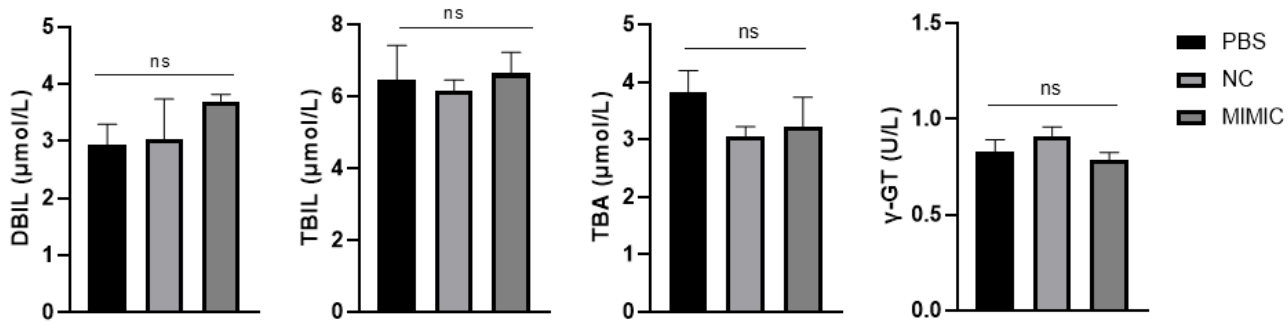

Supplemental Table

A Blood routine indexes

PBS

Abbreviation	Results	Per unit	Reference range
WBC	74.2	10^9/L	0.8-10.6
Lymph#	13.1	10^9/L	0.6-8.9
Mon#	3.7	10^9/L	0.04-1.4
Gran#	57.4	10^9/L	0.23-3.6
Lymph%	17.6	%	40-92
Mon%	5.1	%	0.9-18
Gran%	77.3	%	6.5-50
RBC	7.60	10^12/L	6.5-11.5
HGB	124	g/L	110-165
HCT	40.8	%	35-55
MCV	53.8	fL	41-55
MCH	16.3	pg	13-18
MCHC	303	g/L	300-360
RDW	13.9	%	12-19
PLT	1064	10^9/L	400-1600
MPV	5.4	fL	4.0-6.2
PDW	16.3		12.0-17.5
PCT	0.574	%	0.100-0.780

Non-light-treated HPDENs

Abbreviation	Results	Per unit	Reference range
WBC	8.2	10^9/L	0.8-10.6
Lymph#	1.9	10^9/L	0.6-8.9
Mon#	0.6	10^9/L	0.04-1.4
Gran#	5.7	10^9/L	0.23-3.6
Lymph%	22.9	%	40-92
Mon%	7.6	%	0.9-18
Gran%	69.5	%	6.5-50
RBC	6.09	10^12/L	6.5-11.5
HGB	94	g/L	110-165
HCT	36.8	%	35-55
MCV	52.3	fL	41-55
MCH	15.4	pg	13-18
MCHC	315	g/L	300-360
RDW	14.5	%	12-19
PLT	716	10^9/L	400-1600
MPV	5.5	fL	4.0-6.2
PDW	16.4		12.0-17.5
PCT	0.393	%	0.100-0.780

Light-treated HPDENs

Abbreviation	Results	Per unit	Reference range
WBC	2.9	10^9/L	0.8-10.6
Lymph#	2.2	10^9/L	0.6-8.9
Mon#	0.1	10^9/L	0.04-1.4
Gran#	4.6	10^9/L	0.23-3.6
Lymph%	74.3	%	40-92
Mon%	6.0	%	0.9-18
Gran%	65.0	%	6.5-50
RBC	8.02	10^12/L	6.5-11.5
HGB	124	g/L	110-165
HCT	40.6	%	35-55
MCV	50.7	fL	41-55
MCH	15.4	pg	13-18
MCHC	305	g/L	300-360
RDW	13.1	%	12-19
PLT	667	10^9/L	400-1600
MPV	5.7	fL	4.0-6.2
PDW	16.7		12.0-17.5
PCT	0.380	%	0.100-0.780

Each group comprised n=3-5 mice. Data are presented as means ± SEM.

Supplemental Table

B Blood routine indexes

PBS

Abbreviation	Results	Per unit	Reference range
WBC	24.1	10^9/L ↑	0.8-10.6
Lymph#	17.2	10^9/L ↑	0.6-8.9
Mon#	1.1	10^9/L	0.04-1.4
Gran#	5.8	10^9/L ↑	0.23-3.6
Lymph%	50.8	%	40-92
Mon%	7.9	%	0.9-18
Gran%	62.3	% ↑	6.5-50
RBC	5.00	10^12/L	6.5-11.5
HGB	106	g/L	110-165
HCT	25.3	%	35-55
MCV	50.7	fL	41-55
MCH	21.2	pg	13-18
MCHC	418	g/L	300-360
RDW	14.0	%	12-19
PLT	378	10^9/L	400-1600
MPV	6.2	fL	4.0-6.2
PDW	17.4		12.0-17.5
PCT	0.234	%	0.100-0.780

NC

Abbreviation	Results	Per unit	Reference range
WBC	27.0	10^9/L ↑	0.8-10.6
Lymph#	23.1	10^9/L ↑	0.6-8.9
Mon#	1.1	10^9/L	0.04-1.4
Gran#	3.8	10^9/L ↑	0.23-3.6
Lymph%	85.7	%	40-92
Mon%	4.1	%	0.9-18
Gran%	50.2	% ↑	6.5-50
RBC	5.63	10^12/L	6.5-11.5
HGB	132	g/L	110-165
HCT	31.9	%	35-55
MCV	46.7	fL	41-55
MCH	13.4	pg	13-18
MCHC	313	g/L	300-360
RDW	13.6	%	12-19
PLT	691	10^9/L	400-1600
MPV	6.0	fL	4.0-6.2
PDW	16.7		12.0-17.5
PCT	0.414	%	0.100-0.780

MIMIC

Abbreviation	Results	Per unit	Reference range
WBC	10.4	10^9/L	0.8-10.6
Lymph#	7.4	10^9/L	0.6-8.9
Mon#	0.7	10^9/L	0.04-1.4
Gran#	5.3 ↑	10^9/L	0.23-3.6
Lymph%	29.1	%	40-92
Mon%	7.9	%	0.9-18
Gran%	63.0 ↑	%	6.5-50
RBC	4.91	10^12/L	6.5-11.5
HGB	119	g/L	110-165
HCT	28.7	%	35-55
MCV	50.5	fL	41-55
MCH	20.2	pg	13-18
MCHC	324	g/L	300-360
RDW	14.0	%	12-19
PLT	649	10^9/L	400-1600
MPV	5.9	fL	4.0-6.2
PDW	17.3		12.0-17.5
PCT	0.382	%	0.100-0.780

Each group comprised n=3-5 mice. Data are presented as means ± SEM.

Table S1 Primers/miRNA sequences

nta-miR-172f mimic (5'-3')	TTGGAACGATACAGAGAA	
Primers	Forward (5'-3')	Reverse (5'-3')
nta-miR-172f	TTGGAACGATACAGAGAA	PROVIDED IN THE KIT
CD11c	CACTCAGTGACTGCCCAAAA	CCTCAAGACAGGACATCGCT
IL-1β	ACTACAGGCTCCGAGATGAACAAC	CCCAAGGCCACAGGTATTTT
CD206	CATGGATGTTGATGGCTACTGGAG	GTCTGTTCTGACTCTGGACACTGG
IL-6	CACATGTTCTCTGGGAAATCG	TTGTATCTCTGGAAGTTTCAGATTGTT
TNF-α	ACGGCATGGATCTCAAAGAC	AGATAGCAAATCGGCTGACG
IL-10	TGTCAAATTCATTCATGGCCT	ATCGATTTCTCCCCTGTGAA
Ym1	AGAGTGCTGATCTCAATGTGG	GGGCACCAATTCCAGTCTTAG
TGF-β1	TGCTAATGGTGGACCGCAA	CACTGCTTCCCGAATGTCTGA
18S	AGTCCCTGCCCTTTGTACACA	CGATCCGAGGGCCTCACTA
